# Does social media usage ameliorate loneliness in rural youth? A cross sectional pilot study

**DOI:** 10.1186/s12888-023-04849-y

**Published:** 2023-05-26

**Authors:** Lachlan Gregory, Tegan Dutton, Uchechukwu Levi Osuagwu, Robyn Vines

**Affiliations:** 1 Present Address: Bathurst Rural Clinical School, School of Medicine, Western Sydney University, Bathurst, NSW 2795 Australia; 2 Translational Health Research Institute (THRI), Western Sydney University, Campbelltown, NSW 2560 Australia; 3 African Vision Research Institute (AVRI), Department of Optometry, University of KwaZulu-Natal, Durban, South Africa

**Keywords:** Loneliness, Psychological distress, Rural, Facebook, Social media, Youth

## Abstract

**Aim:**

To investigate the relationship between social media use and loneliness and psychological wellbeing of youth in rural New South Wales.

**Design:**

This was a web-based cross-sectional survey.

**Methods:**

The survey consisted of 33 items including demography (12 items), participants’ social media use (9 items), mood and anxiety (6 items), perceived loneliness (6 items), the impact of COVID-19 on social media usage or perceived loneliness (2 items). The participants’ mood and anxiety were evaluated using the psychological distress tool (K6), while loneliness was measured using the De Jong Gierveld 6-item scale. Total loneliness and psychological distress scores were compared between demographic variables.

**Results:**

A total of 47 participants, aged 16–24 years took part in the study. The majority were women (68%) and many had K6 score that was indicative of psychological distress (68%). About half of the participants indicated that Facebook (FB) was their most used social media platform and two in five participants were on social media within 10 min of waking up each day, about 30% spent more than 20 h per week on social media, and more than two-third sent private messages, images, or videos, multiple times a day. The mean loneliness score was 2.89 (range, 0 to 6), with 0 being ‘not lonely’ and 6 being ‘intense social loneliness’. One-way ANOVA and χ2 test results showed that those who used FB most frequently had significantly higher mean scores for loneliness compared to those that used other social media platforms (*p* = 0.015). Linear regression analysis revealed that those who commonly used FB were more likely to report higher loneliness scores (coefficient = –1.45, 95%CI –2.63, –0.28, *p* = 0.017), while gender (*p* = 0.039), age (*p* = 0.048), household composition (*p* = 0.023), and education level (*p* = 0.014) were associated with severe psychological distress.

**Conclusions:**

The study found that social media usage, particularly FB, as measured by time used and active or passive engagement with the medium, was significantly linked to loneliness, with some impact on psychological distress. Social media use within ten minutes of waking increased the likelihood of psychological distress. However, neither loneliness nor psychological distress were associated with rurality among the rural youth in this study.

**Supplementary Information:**

The online version contains supplementary material available at 10.1186/s12888-023-04849-y.

## Introduction

Human connectedness can be experienced as an innate human need, alongside other more recognised needs like food and water [1]. The feeling of loneliness is the signal that the individual's need for human connection is not being met, much like hunger and thirst are signals for food and water. Loneliness has been described as the experienced discrepancy between the individual’s perceived state of social connection and their desired state of social connection [2, 3]. Loneliness is subjective and borne from the subject’s idea of what necessary connection is. The subjective experience of loneliness accounts for people feeling alone with people around them and vice versa. The disconnect between the perceived and desired connection resulting in loneliness has been shown to negatively impact an individual's well-being and has gained attention as a growing public health issue [1–3]. This is different to isolation which refers to the objective lack of connection a person may have (number of relatives, friends, colleges etc.) due to factors such as geography, education, lifestyle or disability [4–6].

Some studies have shown an association between loneliness and increased all-cause mortality, increased risk of premature mortality and increased risk of chronic morbidities including cardiovascular disease, chronic hypertension, stroke, and cognitive decline [6–9]. Furthermore, loneliness is also associated with increased levels of anxiety, suicidal ideation and behaviours, and a decreased tendency to utilise healthcare.^8^ It is acknowledged though that among the rapidly rising volumes of literature surrounding the topic, not all studies have been able to demonstrate such a relationship between loneliness and mortality. Studies that evaluated both social isolation and loneliness found that social isolation better explained the difference in mortality [1, 10–12]. These differences may in part be accounted for by differing methodologies and ways of assessing the subjective variable of loneliness and comparing it to the objective variable of social isolation.

Previous research has shown that individuals in rural areas generally reported less loneliness than those living in urban areas [5, 13–15]. However, rural youth have been found to have a specific susceptibility to loneliness due to their greater degree of social isolation than their metropolitan counterparts [5]. Rural youth face many challenges due to increased susceptibility to social isolation, decreased availability of health services and poorer outcomes of mental health conditions in rural areas [16, 17]. Although prevalence has been found to be similar, rural areas have poorer mental health outcomes compared to urban areas, resulting in more disabling and socially isolating forms of mental health conditions [16, 17]. Mental health conditions have been shown to be associated with an increase in perceived loneliness, partially due to the decreased ability to form social connections and can therefore be considered a socially isolating factor if disabling enough [15].

It is predicted that individuals who spend more time on social media may manifest greater degrees of distress, as reported in the literature, and that those who are passive users will report greater distress than active users [3, 9]. It is also expected that low-to-moderate (less than 4 h) active usage of social media will have an inverse relationship to loneliness and promote social connectedness. Youth reporting higher levels of distress are expected to report higher levels of loneliness. Overall, the expectation is that social media use is beneficial as a supplement to off-line engagement but is not able to completely substitute for that experience in preventing loneliness [18].

Similar pilot studies [19–22] from as little as 20 participants [20] have been conducted in countries. These studies showed a variance in loneliness and social media use in different regions, as well as create and validate possible interventions that may alleviate loneliness. These pilot studies are a necessity for the preliminary understanding of loneliness in specific subgroups, which will go on to inform further effective studies and interventions.

It has been increasingly asserted that several countries are experiencing an epidemic of loneliness [23]. It is therefore important that, just as research continues in relation to other behavioral risk factors such as obesity or smoking, to provide compelling evidence base relevant to loneliness may help to inform medical practice and community education programmes. This research sought to explore the effect social media use had on alleviating loneliness, by comparing loneliness to duration and engagement SMU and psychological distress.

## Methods

### Study design and population

This pilot study was a web-based cross-sectional survey conducted from October 2021 and October 2022. The participants were those aged between 16—24 years old, and who reported living in a NSW rural area for more than one month.

### Questionnaire design

The survey included incorporating both closed and open-ended questions and supplementary Table (STable-1) presents a sample of the questionnaire including the demography (12 items), participants’ social media use (9 items), mood and anxiety (6 items), and perceived loneliness (6 items). The final two questions addressed the possible impact that COVID-19 may have had on either their social media usage or their perceived loneliness. Out of the 23 questions (excluding demographics, which were a combination of multiple choice and text depending on answers given), nineteen questions were multiple choice.

The questions concerning social media use were developed using a multidimensional Facebook scale which was modified to include social media modalities outside of Facebook, and to clarify the participants’ active or passive engagement with the platform [3]. The items in the survey were shown to be related to loneliness [24]. The participants’ mood and anxiety were evaluated using the K6, an abridged version of the K10 which is a simple instrument designed to measure psychological distress, that had demonstrated benefits from brevity and consistency in screening for mood or anxiety disorders [2]. The loneliness component was constructed using the De Jong Gierveld 6-item scale, a validated assessment tool developed specifically to measure loneliness [25].

This self-administered survey was reviewed by the research team, with variations in grammar, wording or presentation being considered to improve clarity, relevance, and appropriateness of answers, thereby making them more suitable for assessment. None of the questions in the survey were made compulsory, although stating that the individual was outside of the relevant age range ended the survey for them. The survey remained active for the user up to a week after the initiation.

### Recruitment

The survey creation and data collection process were conducted using Qualtrics software (version 2020 of Qualtrics, Provo, Utah, USA). The survey which was targeted at rural youth (aged 16 -24) [26] was circulated to various rural communities across New South Wales. The primary methods of distribution were through paper flyers with the QR code attached or through Facebook (FB) forum posting. Emails, and in-person pamphlets were also sent to various youth organizations and universities requesting further promotion of the survey, while posters with QR codes were posted on various community social media platforms and on notice boards at public places. A detailed list of survey distribution areas can be found in Appendix 1. Following the low response rate, the post was also ‘boosted’ through FB's advertising support for a total of 28 days. Ethics was sought and approved by the Human Research Ethics Committee of Western Sydney University (#: xxxxxxxx). The study was conducted in accordance with the Tenets of the Declaration of Helsinki and all participants provided online consent prior to completing the survey. Ethics did not cover access to NSW high schools.

### Statistical analysis

The data was cleaned in excel. There were 98 raw responses. After removing responses that did not contribute past question 12 or did not respond to components of the K6 or loneliness scale (questions 20—31) which are sensitive to completion, only 47 responses remained. The total loneliness scores consisted of the social (items 24, 25 and 27; responses structured into half the time, somewhat, never) and emotional loneliness scores (items 23, 26 and 28; responses structured as half the time, often, always). For social loneliness scores, a point was given for neutral or negative answers and for emotional loneliness scores, points were given for neutral and positive answers. For analysis, the K6 scores ranged from ‘one’ (minimum score) for ‘none of the time’ to five (maximum score) for ‘all of the time’. The generated total scores were used to categorize into two groups in line with previous study [27, 28] with 6–18 indicating no probable serious mental illness and 19–30 indicating probable serious mental illness.

Rurality was categorized according to the Modified Monash Model classification system. It determines 7 classes of remoteness of Australian towns and cities according to population and distance to services (Government, 2019). For analysis these codes were then grouped into metropolitan (MM1), regional (MM2—MM3) and remote (MM4—MM7).

The loneliness and psychological distress scores were calculated for each independent variable and presented descriptively. Total loneliness and psychological distress scores were compared for each independent variable characteristic using one way analysis of variance (ANOVA) and χ2 test of association, respectively. The analysis was run using Jamovi [29]. *P* < 0.05 was considered statistical significance. Multiple linear regression (MLR) and binomial logistic regression (BLR) were conducted to assess the factors associated with loneliness and psychological distress respectively.

### Data exclusion

To enable scoring, only data for participants who completed the first 33 items of the survey (excluding the 2 final items concerning the impact of COVID-19) were included in the analysis. All results that were incomplete or from a non-NSW and/or non-rural area were excluded.

## Results

### Demographic characteristics and social media use

Table 1 displays the sociodemographic characteristics of the 47 individuals from NSW who participated in the study as well the patterns of social media use, loneliness and the K6 scores. The majority were women (68.1%) and more than half of them were aged 21 or above (56.1%).

**Table 1:** Sociodemographic, social media use and main outcome of the study sample. (*n* = 47, otherwise stated)

Demographics	Frequency (%)
**Age (*n* = 41)**	
16—20	18 (43.9)
21—24	23 (56.1)
**Gender**	
Male	15 (31.9)
Female	32 (68.1)
**Rurality (*n* = 45)**	
Metropolitan	15 (33.3)
Regional	26 (57.8)
Remote	4 (8.9)
**Household composition**	
Lives alone	7 (14.9)
Lives with other, not parents	19 (40.4)
Lives with parents	21 (44.7)
**Employment status**	
Unemployed	9 (19.1)
< 20 h of employment per week	16 (34.0)
> 20 h of employment per week	22 (46.8)
**Education level**	
Certificate or diploma	21 (44.7)
Graduate studies	26 (55.3)
**Social media factors**	
**Time spent using social media per week (*n* = 44)**	
0—10 h	17 (38.6)
11—20 h	12 (27.3)
21—30 h	9 (20.5)
> 30 h	6 (13.6)
**Facebook as most used social media (*n* = 46)**	
Yes	22 (47.8)
No	24 (52.2)
**Use within 10 min of waking**	
Daily	19 (40.4)
A few times per week	20 (42.6)
Once per week	0 (0.0)
A few times per month	5 (10.6)
Never	3 (6.4)
**How often do you send a private message, image, or video on social media?**	
Multiple times a day	36 (76.6)
Multiple times a week	7 (14.9)
Multiple times a month	2 (4.3)
Less than once a month	2 (4.3)
**How often do you post a public message, image or video on social media?**	
Multiple times a day	2 (4.3)
Multiple times a week	8 (17.0)
Multiple times a month	19 (40.4)
Less than once a month	18 (38.3)
**How often do you look at your friends' profiles or social media accounts?**	
Multiple times a day	9 (19.1)
Multiple times a week	14 (29.8)
Multiple times a month	13 (27.7)
Less than once a month	11 (23.4)
**How often do you browse profiles or social media accounts of people you do not know?**	
Multiple times a day	8 (17.0)
Multiple times a week	14 (29.8)
Multiple times a month	10 (21.3)
Less than once a month	15 (31.9)
**How often do you post content other than pictures such as links, games, news or webpages?**	
Multiple times a day	2 (4.3)
Multiple times a week	3 (6.4)
Multiple times a month	13 (27.7)
Less than once a month	29 (61.7)
**Main outcomes**	
**K6 scores—Psychological distress**	
< 19 (mild-moderate)	15 (31.9)
19 + (severe distress)	32 (68.1)
**Loneliness scores**	
Social loneliness^a^	
0	14 (29.8)
1	11 (23.4)
2	7 (14.9)
3	15 (31.9)
Emotional loneliness^a^	
0	13 (27.7)
1	13 (27.7)
2	10 (21.3)
3	11 (23.4)

^a^0 = not lonely and 6 = intensely lonely

**Fig. 1:**
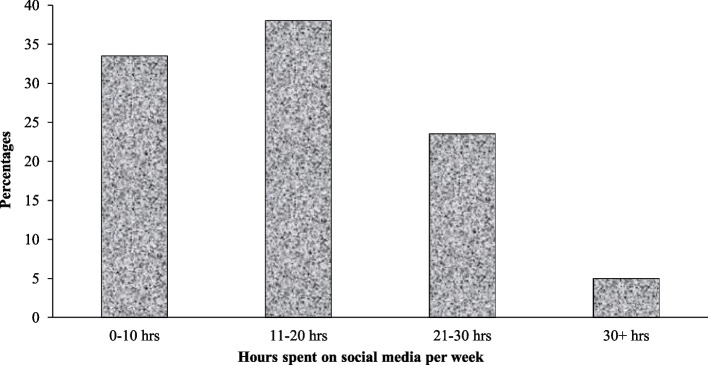
Hours of use of social media amongst those who preference FB as their most common SMU platform

About 50% of the participants indicated that FB was their most used social media platform, with the other 52.2% preferring other social media platforms like TikTok, Snapchat, Instagram, WeChat, Twitter and YouTube. Two in five participants were on social media within 10 min of waking up each day, a little over 30% spent more than 20 h per week on social media, and a majority (76.6%) of them sent private messages, images, or videos, multiple times a day. Figure 1 describes the number of hours the participants spent on social media per week. From the figure, about a quarter of the participants spent more than the three-hour per day threshold which is generally considered the suggested cut-off for all social media users.

### Evaluation outcomes

The mean loneliness score was 2.89 (range, 0 to 6), with 0 being ‘not lonely’ and 6 being ‘intense social loneliness’ [27]. Social loneliness, the indicator of broader engagement with social networks, was found to be slightly more prevalent than emotional loneliness, the indicator of intimate relationships [27].

One-way ANOVA and χ2 analysis results (Table 2) showed that those who reported that FB was their most frequently used social media platform had significantly higher mean scores for loneliness compared to those that used other social media platforms (*p* = 0.015). The scores for loneliness increased with the hours of employment of the participants but this was approaching significance (*p* = 0.069). Rurality, general social media use, time spent on social media, active or passive usage of the preferred social media platform had no influence on the loneliness score.

**Table 2:** Loneliness and psychological distress according to sociodemographic characteristics and SMU (one-way ANOVA and χ2 statistics respectively were used)

Characteristics	Loneliness			Psychological distress	
	Mean (SD)	*P*-value	F (df1, df2)	N (%)	*P*-value
**Age (years)**					
16—20	3.50 (2.07)	0.113	2.65 (1,35.1)	12 (41.4)	0.613
21—24	2.48 (1.90)			17 (58.6)	
**Sex**					
Male	2.33 (1.99)	0.189	1.82 (1,26.0)	7 (21.9)	0.031
Female	3.16 (1.87)			25 (78.1)	
**Rurality**					
Metropolitan	3.52 (1.68)	0.359	1.16 (2,8.19)	9 (30.0)	0.314
Regional	2.62 (1.98)			17 (56.7)	
Remote	3.25 (2.22)			4 (13.3)	
**Household composition**					
Lives alone	2.71 (1.50)	0.677	0.39 (2,19.5)	3 (9.4)	0.212
Lives with other, not parents	3.21 (2.04)			15 (46.9)	
Lives with parents	2.67 (1.98)			14 (43.8)	
**Employment status**					
Unemployed	2.11 (1.45)	0.069	3.00 (2,23.4)	6 (18.8)	0.115
< 20 h of employment per week	2.44 (2.10)			8 (25.0)	
> 20 h of employment per week	3.55 (1.82)			18 (56.3)	
**Education level**					
Certificate or diploma	3.19 (2.02)	0.352	0.88 (1,41.3)	13 (40.6)	0.414
Graduate studies	2.65 (1.85)			19 (59.4)	
**Time spent using social media**					
0—10 h	2.71 (1.93)	0.919	0.16 (3,16.7)	11 (35.5)	0.882
11—20 h	3.08 (1.93)			9 (29.0)	
21—30 h	3.00 (2.40)			7 (22.6)	
> 30 h	2.50 (1.76)			4 (12.9)	
**Facebook as most used social media**					
Yes	3.59 (1.99)	0.015	6.45 (1,41.1)	17 (53.1)	0.277
No	2.21 (1.67)			15 (46.9)	
**Use within 10 min of waking**					
Daily	3.00 (1.00)	0.564	0.72 (3,8.67)	8 (25)	0.012
A few times per week	4.20 (2.05)			16 (50)	
Once per week	0			0 (0.0)	
A few times per month	2.65 (1.73)			5 (15.6)	
Never	2.79 (2.18)			3 (9.4)	
**Passive social media use**					
**How often do you send a private message, image, or video on social media?**					
Multiple times a day	4.00 (2.83)	0.293	2.16 (3,2.57)	26 (81.3)	0.118
Multiple times a week	5.00 (1.41)			4 (12.5)	
Multiple times a month	4.00 (1.53)			2 (6.3)	
Less than once a month	2.50 (1.86)			0 (0.0)	
**How often do you post a public message, image, or video on social media?**					
Multiple times a day	2.61 (1.91)	0.287	1.67 (3,5.01)	12 (37.5)	0.488
Multiple times a week	3.53 (1.93)			14 (43.8)	
Multiple times a month	2.50 (1.77)			4 (12.5)	
Less than once a month	1.00 (1.41)			2 (6.3)	
Active social media use					
**How often do you look at your friends' profiles or social media accounts?**					
Multiple times a day	2.55 (1.75)	0.715	0.45 (3,22.9)	7 (21.9)	0.064
Multiple times a week	3.38 (1.89)			8 (25.0)	
Multiple times a month	2.86 (2.25)			12 (37.5)	
Less than once a month	2.67 (1.80)			5 (15.6)	
**How often do you browse profiles or social media accounts of people you do not know?**					
Multiple times a day	3.60 (1.80)	0.189	1.74 (3,21.2)	7 (21.9)	0.616
Multiple times a week	3.00 (1.76)			9 (28.1)	
Multiple times a month	2.71 (2.05)			6 (18.8)	
Less than once a month	1.75 (1.83)			10 (31.3)	
**How often do you post content other than pictures such as links, games, news, or webpages?**					
Multiple times a day	2.90 (1.97)	0.489	0.98 (3,3.67)	2 (6.3)	0.442
Multiple times a week	3.23 (1.79)			3 (9.4)	
Multiple times a month	2.67 (2.52)			8 (25.0)	
Less than once a month	1.00 (1.41)			19 (59.4)	

The K6 scores were indicative of psychological distress in 68.1% of the participants (Table 1). The proportion with severe psychological distress was significantly higher in females than males (78.1 vs 21.9%, *p* = 0.031) and more in those who frequently used social media within 10 min of waking up compared with other groups (*p* = 0.012, Table 2).

### Factors associated with loneliness and severe psychological distress

The adjusted odd ratios and their 95% CI for factors associated with loneliness and severe psychological distress as determined from MLR and BLR are presented in Tables 3 and 4, respectively. The analysis revealed that compared to those who did not use Facebook, those who commonly used Facebook were more likely to report higher loneliness scores (Coefficient = -1.45, 95%CI -2.63, -0.28, *p* = 0.017; Table 3), No other variable was associated with the loneliness score. From the BLR, we found four characteristics that were significantly associated with severe psychological distress in this study including gender (*p* = 0.039), age (*p* = 0.048), household composition (*p* = 0.023), and education level (*p* = 0.014).

**Table 3:** Multiple linear regression of factors associated with loneliness

Variables	Coefficient	95%CI Lower	*p*
**Gender**	0.87	[-0.40, 2.14]	0.17
**Age**	-1.14	[-2.3, 0.05]	0.06
**Rurality**	-0.33	[-1.41, 0.74]	0.53
**Household composition**	-0.24	[-1.27, 0.78]	0.63
**Employment status**	0.60	[-0.197, 1.39]	0.14
**Educational level**	-0.88	[-2.13, 0.38]	0.17
**How would you rate your general state of health?**	-0.29	[-1.04, 0.45]	0.43
**Most commonly used Facebook**	-1.26	[-2.39, -0.12]	0.03

**Table 4:** Binomial logistic regression (BLR) of factors associated with psychological distress^a^

Predictor	Estimate	Z	*p*
**Gender**	2.57	2.07	0.039
**Age**	3.40	1.98	0.048
**Rurality**	1.94	1.92	0.054
**Household composition**	3.04	2.27	0.023
**Employment status**	1.27	1.59	0.113
**Educational level**	4.19	2.46	0.014
**General State of Health Rating**	0.802	1.253	0.210
**Most commonly used Facebook**	-2.86	-1.74	0.082

^*a*^ Estimates represent the log odds of "Psychological distress = 1" vs. "Psychological distress = 0"

## Discussion

### Principal results

The present study examined loneliness and its association with social media use among rural NSW youth and found no significant correlation between loneliness and the frequency or mode of social media use. However, the degree of loneliness was increased by 1.5 times among those who most commonly used Facebook (FB), which was cited as the most used social media platform by 45% of the participants. The use of other platforms such as Instagram, WhatsApp, Twitter, Snapchat, YouTube or others showed no significant association with loneliness scores. Psychological distress was common among female participants, those who completed a graduate degree, people not living alone and those above 20 years of age. Although over 90% of youth use social media as a daily practice and primary form of communication, there is still contention about whether social media is an effective tool for establishing or maintaining meaningful connection [30]. Some research demonstrates detrimental effects of social media usage and relationships in comparison to the perceived more intimate face-to-face connections, whilst others defend the legitimacy of socialization through a digital medium in the modern world [30]. Both viewpoints can be defended due to the multifactorial and complex nature of how individuals relate and create relationships. Rapid changes in the social media scene, as it tries to better emulate and facilitate meaningful connection, also adds to the variance of results being published.

The higher likelihood of loneliness among those who commonly used FB was surprising. This is because FB offers a mix of active and passive content, with the ability to both publicly post and message individuals or groups of contacts directly. In contrast, platforms such as YouTube or Reddit lend themselves to being more public and browsing oriented, with discussion being more forum based. As such, with the mixed methods that FB offers, and its historical place in social media, it was presumed that it would decrease loneliness rather than contribute to it. A potential clarification that would help draw further implications from this would be the number of hours individuals used the FB app or the subsidiary Messenger app. The latter is primarily focused on private active users, and it may help to separate the two.

Past studies suggest that social media has a negative impact on self-image and mental health, but this was dependent on the number of hours spent per day on these apps [31, 32]. Among teenagers in the USA, those that spent more than 3 h a day on social media reported a 60% higher risk of mental health problems compared to those that did not engage with the social media app at all [32]. This led to the suggestion that teenagers should not spent more than 3 h per day on social media. Among adults, researchers found that limiting the amount of time spent per day on social media to 30-min led to a reduction in loneliness, anxiety, depression, and stress scores, compared to spending more than 30 min a day on social media [31]. The nullification of the significant association between social media use and loneliness with the inclusion of hours spent per week on social media, can be attributed to the finding that most people in our study spent less than 3 h a day on social media (70% spent between 0 and 20 h per week), which was below the risk threshold number of hours per day.

Being female was associated with higher levels of psychological distress in this sample population. This was in agreement with past studies, though it is acknowledged that there is an imbalance in the gender of participants that is skewed towards women (68%) [33–35]. Studies have attributed this association to either a sex-based bias in K6 responses or a sex-based difference, potentially attributable to gender roles and social influences [36]. Age greater than 20 years, completing or having completed a graduate level course and not living alone were also significantly associated with loneliness. It is possible that these three variables relate and are natural correlates of each other, for example it would be more likely for the individual to be greater than 20 years of age and involved in a graduate course than under the age of 16. Level of education was the most significant positive predictor of psychological distress, so potentially had the greatest impact of the 3 variables. Possibly contextual reasons for the association between psychological distress and education status includes the changes in education that came with COVID-19, including increased course loads, online learning, restricted interaction and activities and other changes to student lifestyle [37]. In regards to social media use within 10 min of waking, further assessment of internet addiction behaviours could assist in making more meaningful sense of its significance in relation to psychological distress [38].

The association between psychological distress and employment status of the participants (*p* = 0.069), mode of social media use (active users, *p* = 0.064), and rurality (*p* = 0.054) were approaching significance in this study. It is possible that these variables would have had a significant association with psychological distress if a larger sample size had been achieved. Although we found some significance in few variables, efforts were made to increase the sample size, including having the survey available for a period of 1 yr, using the commonly available social media platforms for survey distribution and boosting the project on FB. Other research has shown employment status to be a significant predictor for loneliness and a bidirectional relationship between the two has also been proposed by some [39]. There have been inconsistent results on the role of active social media use, with some studies showing an association with ill-being and others well-being [40–42]. Rural Australian populations have also previously been linked to increased psychological distress, primarily in the context of decreased access to services [43].

## Limitations and strengths

As this was a self-report cross-sectional survey, causality cannot be inferred between social media use and perceived loneliness. A longitudinal design could help assess changes in social media use, loneliness, and psychological wellbeing over time. Few participants took part in the study resulting in the small sample size, which reduces the power to detect significant differences. Although the survey was reported to have a wider reach (5000 plus individuals) according to distribution statistics provided by FB, the number of survey responses did not meet the initial target of 289 responses and some participant responses (*n* = 51 responses) were incomplete and could not be used for analysis. Despite this, the data is useful in adding to the high variance picture of this rapidly evolving area of social media use and loneliness. In addition, our study adds the specific sub-population of Australian rural youth to those under consideration [30, 41, 42]. Previous pilot studies have similarly used smaller *n* = 20 [20], or inadequate samples *n* = 42 [21] and *n* = 72 [19]. The lack of survey response uptake was an unexpected challenge considering the wide recruitment strategies implemented but may suggest an unwillingness for people to partake in research of such sensitivity. Despite numerous attempts to increase response rates over the collection period, only few of these measures were effective. One problem that was encountered during distribution was that the posts onto digital community noticeboards were sometimes removed by the page administrators, due to not being of local origin or deemed relevant. This made it difficult to access some target groups that we would have liked to reach. Although web surveys are time and cost effective, they can suffer from sampling bias, such as reaching people of lower educational status [44]. Also, the study did not collect data on other factors that may influence loneliness and psychological distress, such as personal life events, socioeconomic status, or other mental health issues.

Despite these limitations, the combined approach of distribution through digital web surveys, emails, and in-person pamphlets was considered a positive approach to improving both the quantity and quality of the data. It is recommended that future studies should consider broadening the age range, to include either those that are older (including the elderly who are an ‘at risk’ population for loneliness) and much younger people to capture school-aged individuals. Young people are perceived by some to be transitioning into adults later in life, so extending the upper limit beyond our age range by 3 to 5 years may be appropriate, whilst still focusing on youth [45]. Other research has also shown that incentives for completing surveys increase response rates, so this may be something to consider, if within the study’s means, subject to funding availability. However, it has been found that the efficacy of incentives differs across sub-populations and as such, may potentially distort the composition of the participant groups [44]. Whilst a strength of this paper was in trying to determine and link the type of social media usage, such as active or passive, to loneliness or psychological distress in rural NSW youth populations, it has been noted that the rapid release of new research concepts and terminology continues to be a challenge e.g. in the questioning of the active–passive paradigm [40]. For future research in this evolving field, use of modern tools and instruments would allow for research to meaningfully test new theories that better explain engagement and connection in the digital world. Another strength of this paper was the targeting of a potentially at-risk population. It is already known that youth and elderly people are the most likely groups to experience loneliness, and that rural populations are at risk due to geographical isolation [16, 17, 46, 47]. Focusing on youth in such studies is important as they are growing up with social media as a primary means of communication, which may connect them differently to those who grow up in an alternative context. Continuous study of future generations will allow for further understanding of trends in the emerging digital world.

## Recommendations

Human connection remains crucial to the health and wellbeing of all people. However, the way in which people seek and develop these connections has changed and will foreseeably continue to change in the future. Successful interventions that promote social connection and decrease loneliness require in-depth understanding of how people effectively connect and how to use this in promoting well-being. Current research findings should be used to advise appropriate use of social media to consumers, especially to children and youth in schooling and home contexts. In addition, further specific research should be undertaken to elicit person-specific SMU effects on health and well-being.

## Conclusion

Our results support some of the current research literature that suggests social media usage, as measured by time used and active or passive engagement with the medium, is for the most part not significantly linked to loneliness or psychological distress. Of significance was the association found between loneliness and Facebook usage as the most common social media platform compared to others, and psychological distress and social media use within ten minutes of waking. Rural populations, due to their relative geographic isolation, were hypothesised to be a population group that would benefit from the increased accessibility to the avenues of connection that social media may provide. Interestingly however, in our rural youth sample, there was no association detected between loneliness or psychological distress and rurality.

## Supplementary Information

**Additional file 1:** **Appendix 1.** Record of distribution of survey on Facebook.

**Additional file 2:** **STable-1. **Sampleof questionnaire used in this study.

## Data Availability

The datasets generated and/or analyzed during the current study are not publicly available due institutional policy but are available from the corresponding author on reasonable request.

## Abbreviations

ANOVA: Analysis of variance
BLR: Binomial logistic regression
COVID-19: Coronavirus diseases 2019
FB: Facebook
MLR: Multiple linear regression
NSW: New South Wales
