# Supplementary material for: Does social media usage ameliorate loneliness in rural youth? A cross sectional pilot study
Source: BMC Psychiatry. 2023 May 26;23:371. doi: 10.1186/s12888-023-04849-y (PMC10214363; doi:10.1186/s12888-023-04849-y)
Supplement: Supplementary file 1 — Additional file 1: Appendix 1. Record of distribution of survey on Facebook. [file 12888_2023_4849_MOESM1_ESM.docx]

**Appendix 1:** Record of distribution of survey on Facebook

| **FB community page** | **Date posted** | | | | | | | |
| --- | --- | --- | --- | --- | --- | --- | --- | --- |
| Batemans Bay | 13-Jan | 28-Mar | 16-Jun | 2-Jul | 10-Jul | 21-Jul | 1-Sep | 14-Sep |
| Bathurst | 13-Jan | 28-Mar | 16-Jun | 2-Jul | 10-Jul | 21-Jul | 1-Sep | 14-Sep |
| Broken Hill | 14-Jan | 28-Mar | 16-Jun | 2-Jul | 10-Jul | 21-Jul | 1-Sep | 14-Sep |
| Byron Bay | 13-Jan | 28-Mar | 17-Jun | 2-Jul | 10-Jul | 21-Jul | 1-Sep | 14-Sep |
| Cooma | 14-Jan | 28-Mar | 17-Jun | 2-Jul | 10-Jul | 21-Jul | 1-Sep | 14-Sep |
| Cowra | 14-Jan | 28-Mar | 16-Jun | 2-Jul | 10-Jul | 21-Jul | 1-Sep | 14-Sep |
| Dubbo | 14-Jan | 28-Mar | 16-Jun | 2-Jul | 10-Jul | 21-Jul | 1-Sep | 14-Sep |
| Goulburn | 14-Jan | 28-Mar | 17-Jun | 2-Jul | 10-Jul | 21-Jul | 1-Sep | 14-Sep |
| Griffith | 13-Jan | 28-Mar | 16-Jun | 2-Jul | 10-Jul | 21-Jul | 1-Sep | 14-Sep |
| Harden | 14-Jan | 28-Mar | 16-Jun | 2-Jul | 10-Jul | 21-Jul | 1-Sep | 14-Sep |
| Jindabyne | 14-Jan | 28-Mar | 16-Jun | 2-Jul | 10-Jul | 21-Jul | 1-Sep | 14-Sep |
| Lithgow | 14-Jan | 28-Mar | 16-Jun | 2-Jul | 10-Jul | 21-Jul | 1-Sep | 14-Sep |
| Moama | 14-Jan | 28-Mar | 16-Jun | 2-Jul | 10-Jul | 21-Jul | 1-Sep | 14-Sep |
| Nowra | 14-Jan | 28-Mar | 17-Jun | 2-Jul | 10-Jul | 21-Jul | 1-Sep | 14-Sep |
| Orange | 13-Jan | 28-Mar | 17-Jun | 2-Jul | 10-Jul | 21-Jul | 1-Sep | 14-Sep |
| Tamworth | 13-Jan | 28-Mar | 17-Jun | 2-Jul | 10-Jul | 21-Jul | 1-Sep | 14-Sep |
| Tumit | 14-Jan | 28-Mar | 16-Jun | 2-Jul | 10-Jul | 21-Jul | 1-Sep | 14-Sep |
| Wagga Wagga | 14-Jan | 28-Mar | 16-Jun | 2-Jul | 10-Jul | 21-Jul | 1-Sep | 14-Sep |
| NSW rural youth |  |  |  |  |  | 11-Aug |  |  |
| PCYC NSW |  |  |  |  |  | 11-Aug |  |  |
| CSU |  |  |  |  |  | 16-Aug | 1-Sep |  |
| Bathurst Young People group |  |  |  |  |  | 16-Aug | 1-Sep |  |
