# Supplementary material for: Does social media usage ameliorate loneliness in rural youth? A cross sectional pilot study
Source: BMC Psychiatry. 2023 May 26;23:371. doi: 10.1186/s12888-023-04849-y (PMC10214363; doi:10.1186/s12888-023-04849-y)
Supplement: Supplementary file 2 — Additional file 2: STable-1. Sampleof questionnaire used in this study. [file 12888_2023_4849_MOESM2_ESM.docx]

## Supplementary file

**STable-1:** Sample of questionnaire used in this study

| 1. **Gender** 2. **Please enter your age (years). If age is outside 16 – 24 years then you will be redirected to the end of the survey.** 3. **Home address postcode** 4. **For how many years have you been living in the district of your current postcode?** 5. **Household composition** 6. Living with parent(s) 7. Living with partner, no children 8. Living with partner and children 9. Living alone 10. Other, please specify 11. **Employment status (paid employment)** 12. Unemployed 13. Employed, 20 or more hours a week 14. Employed, <20 hours a week 15. Other, please specify 16. **Are you currently studying:** Yes / No      1. **If yes, what level are you currently studying:** 2. School certificate 3. Higher school certificate 4. Diploma/certificate 5. Undergraduate degree 6. Postgraduate degree 7. **If no, what is the highest level of education you have completed** 8. School certificate 9. Higher school certificate 10. Diploma/Certificate 11. Undergraduate degree 12. Postgraduate degree 13. **How would you rate your general state of health?** 14. Poor 15. Not so good 16. Fair 17. Good 18. Very good 19. **Please list the top 3 social media platforms that you use (with 1 being the most common):**   23. **On average, how many hours per week do you spend on social media?** 24. **On average, how many days per week do you use social media?** 25. **How often do you use social media within 10 minutes of waking up?** 26. Daily 27. A few times per week 28. Once per week 29. A few times per month 30. Never 31. **How often do you send a private message, image or video on social media?** 32. 6 times per day or more 33. 2 to 5 times per day 34. Once per day 35. A few times per week 36. Once per week 37. A few times per month 38. Once a month or less 39. Never 40. **How often do you post a public message, image or video on social media?** 41. 6 times per day or more 42. 2 to 5 times per day 43. Once per day 44. A few times per week 45. Once per week 46. A few times per month 47. Once a month or less 48. Never 49. **How often do you look at your friends' profiles or social media accounts?** 50. 6 times per day or more 51. 2 to 5 times per day 52. Once per day 53. A few times per week 54. Once per week 55. A few times per month 56. Once a month or less 57. Never      1. **How often do you browse profiles or social media accounts of people you do not know?** 2. 6 times per day or more 3. 2 to 5 times per day 4. Once per day 5. A few times per week 6. Once per week 7. A few times per month 8. Once a month or less 9. Never | **19**. **How often do you post content other than pictures such as links, games, news or webpages?**   1. 6 times per day or more 2. 2 to 5 times per day 3. Once per day 4. A few times per week 5. Once per week 6. A few times per month 7. Once a month or less 8. Never   **20**. **How often do you feel nervous?**   1. Always 2. Most of the time 3. About half the time 4. Sometimes 5. Never   **21**. **How often do you feel hopeless?**   1. Always 2. Most of the time 3. About half the time 4. Sometimes 5. Never   **22**. **How often do you feel restless or fidgety?**   1. Always 2. Most of the time 3. About half the time 4. Sometimes 5. Never   **23**. **How often do you feel so depressed that nothing could cheer you up?**   1. Always 2. Most of the time 3. About half the time 4. Sometimes 5. Never   **24**. **How often do you feel everything was an effort?**   1. Always 2. Most of the time 3. About half the time 4. Sometimes 5. Never   **25**. **How often do you feel worthless?**   1. Always 2. Most of the time 3. About half the time 4. Sometimes 5. Never   **26**. **How often do you experience a general sense of emptiness?**   1. Always 2. Most of the time 3. About half the time 4. Sometimes 5. Never   **27**. **Are there plenty of people you can rely on when you have a problem?**   1. Always 2. Most of the time 3. About half the time 4. Sometimes 5. Never   **28**. **Are there plenty of people you can trust completely?**   1. Always 2. Most of the time 3. About half the time 4. Sometimes 5. Never   **29**. **How often do you miss having people around?**   1. Always 2. Most of the time 3. About half the time 4. Sometimes 5. Never   **30**. **How often do you feel there are enough people close to you?**   1. Always 2. Most of the time 3. About half the time 4. Sometimes 5. Never   **31**. **How often do you feel rejected?**   1. Always 2. Most of the time 3. About half the time 4. Sometimes 5. Never   **32**. **How would you describe the impact of the COVID-19 pandemic on your social media use?**  **33**. **Any other comments you would like to make about your use of social media since the COVID-19 pandemic began?** |
| --- | --- |
